# SOFA score and short-term mortality in acute decompensated heart failure

**DOI:** 10.1038/s41598-020-77967-2

**Published:** 2020-11-30

**Authors:** Adi Elias, Reham Agbarieh, Walid Saliba, Johad Khoury, Fadel Bahouth, Jeries Nashashibi, Zaher S. Azzam

**Affiliations:** 1 Internal Medicine Department B, Rambam Health Care Campus, PO Box 9602, 31096 Haifa, Israel; 2 Bruce and Ruth Rappaport Faculty of Medicine, Technion – Israel Institute of Technology, Haifa, Israel; 3 Department of Community Medicine and Epidemiology, Lady Davis Carmel Medical Center, Haifa, Israel; 4 Pulmonology Division, Lady Davis Carmel Medical Center, Haifa, Israel; 5 Cardiology Department, Rambam Health Care Campus, Haifa, Israel; 6 Internal Medicine Department H, Rambam Health Care Campus, Haifa, Israel; 7 Medicine Department D, Rambam Health Care Campus, Haifa, Israel

**Keywords:** Cardiology, Health care, Medical research, Risk factors

## Abstract

Acute decompensated heart failure (ADHF) is one of the leading causes for hospitalization and mortality. Identifying high risk patients is essential to ensure proper management. Sequential Organ Function Assessment Score (SOFA) is considered an excellent score to predict short-term mortality in sepsis and other life-threatening conditions. To assess the capability of SOFA score in predicting short-term mortality in ADHF. We retrospectively identified patients with first hospitalization with primary diagnosis of ADHF between the years (2008–2018). The SOFA score was calculated for all patients. A total 3232 patients were included in the study. The SOFA score was significantly associated with in-hospital mortality and 30-day mortality. The odds ratios for 1-point increase in the SOFA score were 1.86 (95% CI 1.68–1.96) and 1.627 (95% CI 1.523–1.737) respectively. The SOFA Score demonstrated a good predictive accuracy. The areas under the curve of receiver operating characteristic curves for in-hospital mortality and 30-day mortality were 0.765 (95% CI 0.733–0.798) and 0.706 (95% CI 0.676–0.736) respectively. SOFA score is associated with increased risk of short-term mortality in ADHF. SOFA can be used as a complementary risk score to screen high risk patients who need strict monitoring.

## Introduction

Heart failure (HF) is a major public health problem, with a prevalence of over 23 million worldwide^1^. Patients hospitalized due to acute decompensated heart failure (ADHF) are at high risk for short- and long-term mortality. Identifying patients with increased risk of mortality is essential to ensure proper monitoring and management^2,3^.

A number of laboratory markers has been associated with increased risk of short term mortality, Blood Urea Nitrogen (BUN) and natriuretic peptides level both in admission and discharge were associated with increased risk for short term mortality^4–7^.

Several risk scores have been developed to predict short-term mortality in ADHF^8^. Some of those risk scores have excellent predictive capability but are impractical in the emergency or critical care setting due to large number of variables^9^, other scores for example are simple but lack good predictive capabilities or based on comorbid conditions^10^.

Sequential Organ Function Assessment Score (SOFA) is considered an excellent score to predict short-term mortality in sepsis and other life threatening conditions^11,12^. According to a recent research, the SOFA score appears to have good predictive capabilities in cardiac intensive units^13,14^. However, patients in those studies were hospitalized for heterogeneous conditions e.g. myocardial infarction, arrhythmia, cardiac arrest. ADHF shares similar pathophysiologic mechanism with sepsis such as; systemic hypoperfusion and inflammatory state that might lead to organ dysfunction that can be assessed by the SOFA score^15,16^; therefore, it is conceivable to hypothesize that the SOFA score is associated with increased mortality risk in patients with ADHF.

The current study aimed to evaluate the ability of the SOFA score to predict short-term mortality in a large cohort of patients admitted to the emergency department with ADHF.

## Methods

### Data collection

Patients with first hospitalization with the primary diagnosis of ADHF, between December 2008 and February 2018, were retrospectively identified using the computerized database of Rambam Health Care Campus. Patients included in the study were hospitalized in cardiac intensive care units, internal medicine or cardiology departments.

Patients with sepsis, infectious disease during hospitalization, acute coronary syndrome, or pulmonary embolism were excluded from the study.

The study was approved by Rambam Health Care Campus Institutional Review Board and Ethics Committee on human research (Approval ID: RMB-12-0477). Due to the retrospective nature of the study, informed consent was waived by the ethics committee. All the methods were performed in accordance with the relevant guidelines and regulations.

Demographic data, comorbid conditions, regular medications, vital signs, mechanical ventilations parameters at admission, echocardiographic data, laboratory data were collected by the MDClone software (mdclone.com).

### Sequential Organ Function Assessment score (SOFA)

The SOFA sub scores were calculated individually according to severity of system impairment (neurologic, cardiovascular, renal, respiratory, coagulation and hepatic), each system received a score that ranges between 0 and 4 according to Supplementary Table S1.

And the sum of the sub scores generated the total SOFA score. Patients were divided into 4 categories by 2 points intervals; Category 1 (0–1 points), Category 2 (2–3 points), Category 3 (4–5 points), Category 4 (6 and above points).

### Glasgow coma scale (GCS)

Since the Glasgow Coma Scale (GCS) is not routinely assessed in ADHF patients and the retrospective nature of the study, the GCS was assessed in the following manner; patients with normal level of consciousness received 15 points (93.7% of the cohort), comatose patients received 6–9 points (3.7% of the cohort), the medical records of the remaining patients with abnormal mental status (2.6% of cohort) were reviewed by experienced physician who classified GCS according to the documented motor, verbal and eye responses at the admission medical records.

### ADHERE score

The ADHERE is an acceptable score to stratify patients admitted with ADHF, it was chosen for comparison because it is simple, practical and is similar to the SOFA, also due to availability of data. The ADHERE score was calculated to all patients according to the classification tree algorithm, using systolic blood pressure, BUN and Creatinine at admission. Patients were stratified by increasing severity; low, intermediate 3, intermediate 2, intermediate 1 and high risk for in-hospital mortality^10^. The prognostic accuracy of the ADHERE was assessed as ordinal variable.

### GWTG-HF score

Get With the Guidelines–Heart Failure Risk Score is a validated score that was developed using the American Heart Association GWTG‐HF program data and predicts in‐hospital mortality in patients with acute heart failure (HF)^17,18^. The risk score is calculated according to the following variables: age, systolic blood pressure, heart rate, BUN, sodium, black race and COPD diagnosis.

### Framingham risk score

The score was calculated using the following variables: age, sex, smoking status, total cholesterol, HDL cholesterol, systolic blood pressure, previous medication for blood pressure^19^.

### Study outcomes

The primary outcomes were in-hospital and 30-day mortality in the different SOFA score categories. Mortality data were retrieved from the Israeli Ministry of Interior affairs, in order to avoid loss to follow-up.

### Statistical analysis

Continuous variables that are normally distributed are summarized with mean ± standard deviation. Categorical variables are presented with frequencies and proportions.

Logistic regression models were used to assess the association between SOFA score and in-hospital mortality. The strength of the association was estimated with odds ratios with 95% confidence interval (CI), with category 1 serving as reference category. Kaplan–Meier curves were used to depict time to death. The predictive value for in-hospital and 30-day mortality of SOFA score was assessed by calculating the area under the curve (AUC) of the receiver operating characteristic (ROC) curve.

Comparison of the predictive accuracy of the SOFA and the other risk scores were assessed by comparing area under the ROC curves, using the test proposed by DeLong et al.^20^.

In order to inspect the calibration of the SOFA score, we calculated the predicted probabilities of in hospital mortality according to the SOFA score using binary logistic regression, the predicted probabilities were binned for 10 deciles in order to perform the calibration plots.

For all analyses, a p value less than 0.05 for the 2-tailed tests was considered statistically significant. All statistical analyses were performed using SPSS 21.0.

The net benefit of using the SOFA score as a clinical decision tool was estimated by decision curve analysis using R version 3.6.1.

Decision curves plot the predicted net benefit of the scores versus risk thresholds. The net benefit at a given decision threshold is defined as the difference between the proportion of true positives and the proportion of false positives where the latter is weighted by the odds of the specific threshold. The decision curve is then created by calculation of the net benefits for all possible thresholds.

## Results

**Figure 1:**
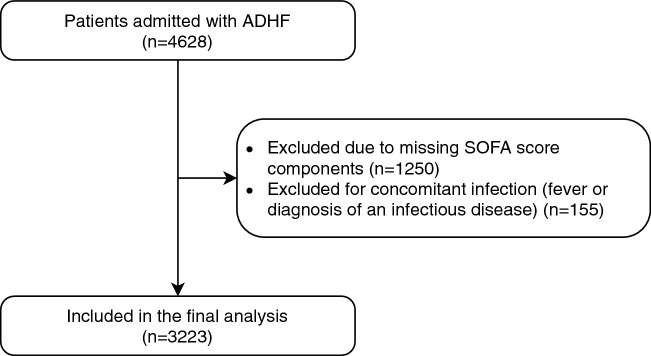
Flow chart of inclusion of acute decompensated heart failure cases in analyses.

**Figure 2:**
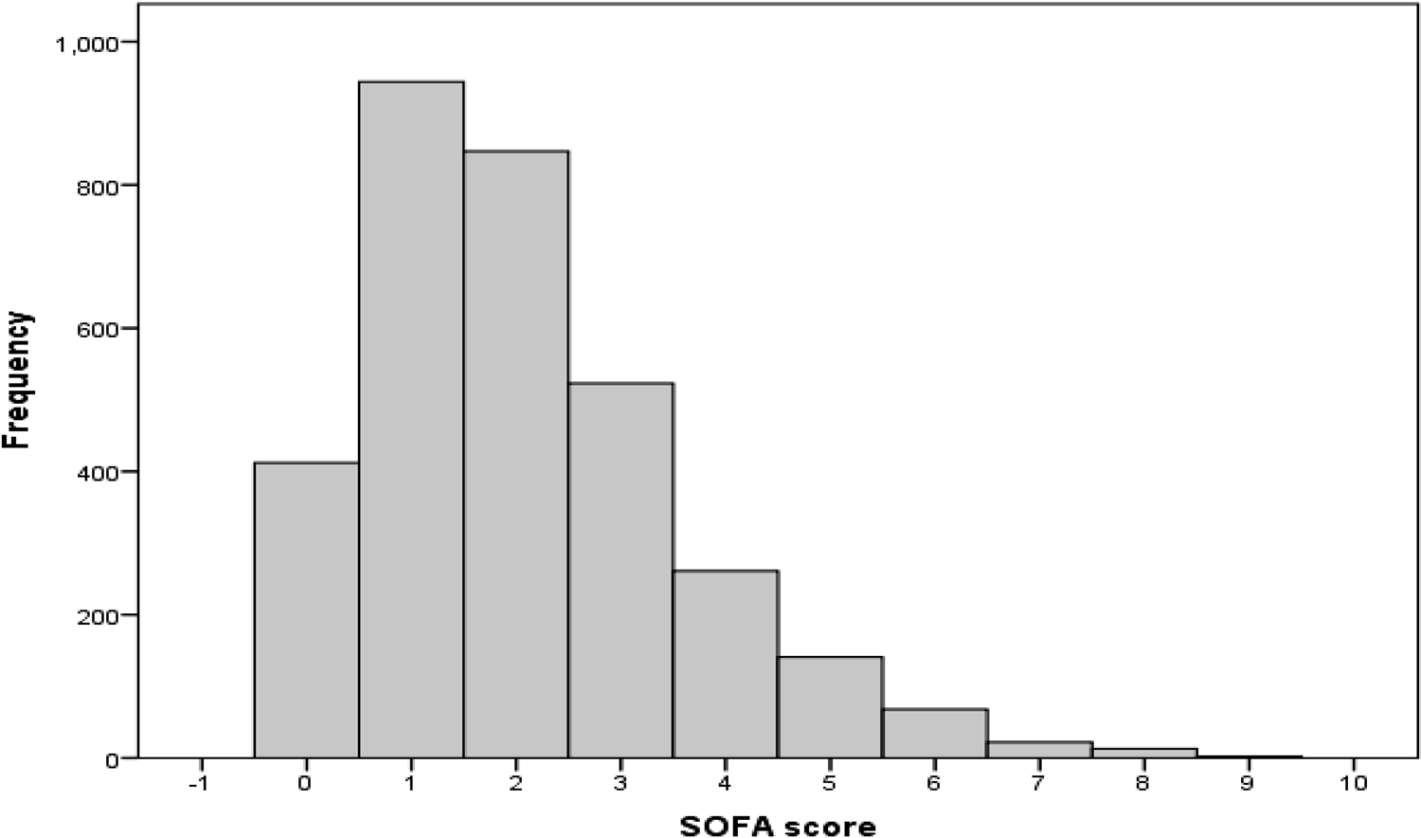
SOFA score frequency distribution in the study cohort.

A total of 3233 patients admitted with ADHF were included in the study Fig. 1. The mean age was 75.15 ± 11.89, and 47.8% were female. The SOFA score distribution and frequency are presented in Fig. 2. The proportion of subjects in each SOFA score category was as follows. Category 1 (41.9%), Category 2 (42.4%), Category 3 (12.4%), Category 4 (3.2%) (Table 1). The demographic data, clinical and echocardiographic characteristics of the study cohort are presented by the SOFA score 4 categories in Table 1.

**Table 1:** Distribution of demographic, clinical characteristics according to SOFA score categories.

	All 3233 (100%)	Category 1 (0–1 points) 1356 (41.9%)	Category 2 (2–3 points) 1370 (42.4%)	Category 3 (4–5 points) 402 (12.4%)	Category 4 (> 5 points) 105 (3.2%)	P Value
Age	75.15 ± 11.89	74 ± 12	76.32 ± 11.5	74.7 ± 12.36	74 ± 11.52	< 0.001
Female Gender	1544 (47.8%)	766 (56.5%)	602 (43.9%)	140 (34.8%)	36 (34.3%)	< 0.001
IHD	1794 (55.5%)	686 (50.6%)	808 (59.0%)	237 (59.0%)	63 (60.0%)	< 0.001
DM	1735 (53.7%)	733 (54.1%)	738 (53.9%)	219 (54.5%)	45 (42.9%)	.161
Hypertension	2696 (83.4%)	1150 (84.8%)	1148 (83.8%)	324 (80.6%)	74 (70.5%)	< 0.001
CKD	886 (27.4%)	216 (15.9%)	465 (33.9%)	164 (40.8%)	41 (39.0%)	< 0.001
COPD	492 (15.2%)	201 (14.8%)	209 (15.3%)	68 (16.9%)	14 (13.3%)	.717
Normal EF (50–70%)	1086 (33.6%)	481 (35.5%)	474 (34.6%)	107 (26.6%)	24 (22.9%)	0.001
Mild EF (40–50%)	293 (9.1%)	126 (9.3%)	120 (8.8%)	43 (10.7%)	4 (3.8%)	0.001
Moderate EF (30–40%)	345 (10.7%)	162 (11.9%)	135 (9.9%)	37 (9.2%)	11 (10.5%)	0.001
Severely Reduced EF (< 30%)	500 (15.5%)	202 (14.9%)	203 (14.8%)	73 (18.2%)	22 (21.0%)	0.001
Missing Echocardiography	1009 (31.2%)	385 (28.4%)	438 (32.0%)	142 (35.3%)	44 (41.9%)	0.001
GFR	51.1 ± 7.4	65 ± 26	43 ± 22.5	35.8 ± 25.4	33.6 ± 3.6	< 0.001
Creatinine	1.57 ± 1	1 ± 0.29	1.7 ± 0.71	2.61 ± 1.75	2.86 ± 2	< 0.001
BUN	32 ± 18.64	22.59 ± 9.13	35.69 ± 17	47.81 ± 23.9	53 ± 30.5	< 0.001
Hemoglobin	11.6 ± 2	11.9 ± 1.9	11.4 ± 2	11.24 ± 2	11.7 ± 2.3	< 0.001
Platelets	299 ± 90	254 ± 85	380 ± 90	201 ± 103	205 ± 100	< 0.001
SOFA	2 ± 1.5	0.69 ± 0.46	2.38 ± 0.48	4.35 ± 0.47	6.5 ± 0.78	< 0.001
Beta Blockers**	2031 (62.8%)	872 (64.3%)	860 (62.8%)	241 (60.0%)	58 (55.2%)	0.15
ACE Inhibitors**	1535 (47.5%)	704 (51.9%)	628 (45.8%)	154 (38.3%)	49 (46.7%)	< 0.001
K sparing**	507 (15.7%)	190 (14.0%)	217 (15.8%)	76 (18.9%)	24 (22.9%)	0.017
Diuretics**	1980 (61.2%)	703 (51.8%)	923 (67.4%)	281 (69.9%)	73 (69.5%)	< 0.001
Length of stay (days)	8.08 ± 8.9	6.32 ± 6.45	8.46 ± 9.47	10.77 ± 10.41	15.54 ± 14.97	< 0.001

**Drugs at admission.

**Figure 3:**
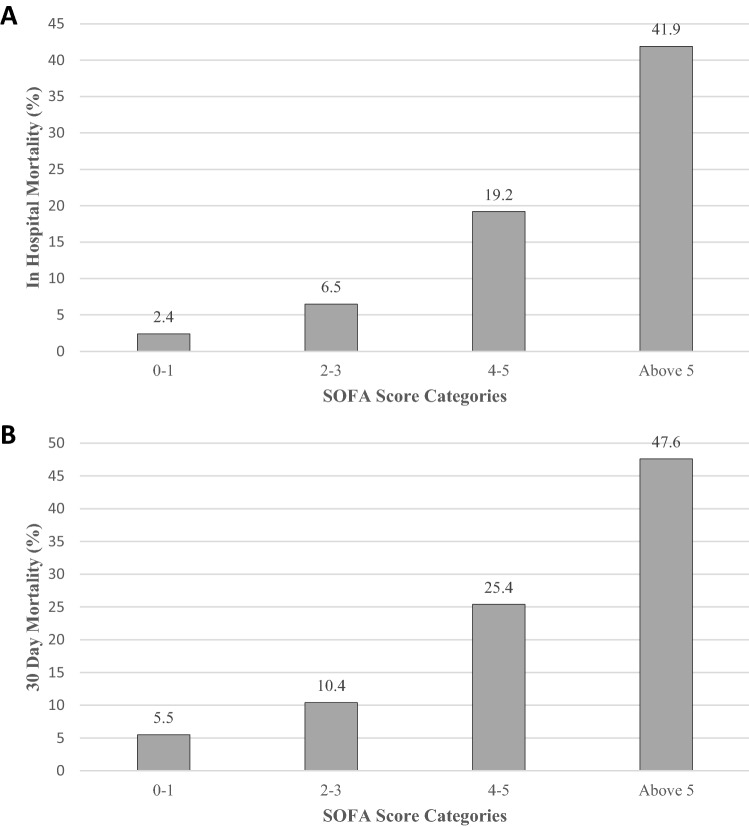
The risk of in-hospital and 30 day mortality according to SOFA score categories.

**Figure 4:**
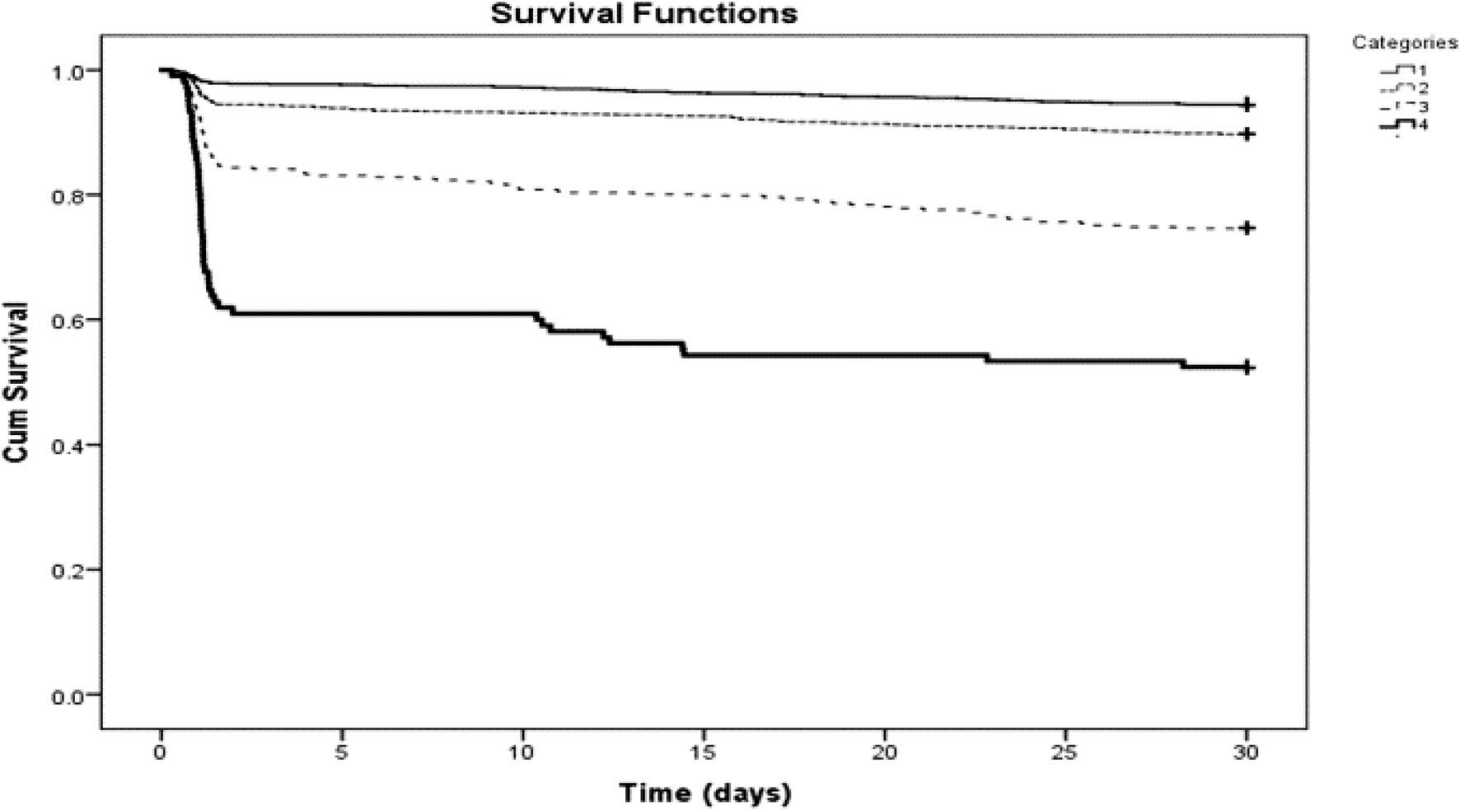
Kaplan–Meier survival curves according to SOFA score categories.

The in-hospital and 30-day mortality risk for the entire study cohort were 7.5% and 11.4% respectively. The risk of in-hospital mortality increased with the severity of the SOFA score; 2.4%, 6.5%, 19.2%, 41.9% for categories 1 through category 4, respectively (P < 0.001) (Fig. 3). The corresponding risk of 30-day mortality were 5.5%, 10.4%, 25.4%, 47.6% respectively (P < 0.001) (Figs. 3 and 4).

The risk for in-hospital mortality increased as the severity of the category increased as shown in Table 2.

**Table 2:** The risk for in-hospital and 30 of death according to SOFA score categories.

System	In Hospital mortality odds ratio (95% CI)	P value	30 Day mortality odds ratio (95% CI)	P value
**SOFA score as categorical variable**				
Category 1 (0–1 points)	Reference	–	Reference	–
Category 2 (2–3 points)	2.875 (1.905–4.337)	< 0.001	1.975 (1.477–2.641)	< 0.001
Category 3 (4–5 points)	9.803 (6.379–15.065)	< 0.001	5.8 (4.2–8.02)	< 0.001
Category 4 (> 5 points)	29.844 (17.695–50.335)	< 0.001	15.527 (9.91–24.3)	< 0.001
SOFA score as continuous variable*				
Crude	1.86 (1.68–1.96)	< 0.001	1.627 (1.523–1.737)	< 0.001
Model**	1.874 (1.719–2.04)	< 0.001	1.657 (1.541–1.781)	< 0.001

*OR for each 1 point increase in SOFA.

**Multivariable model adjustment for Age, Gender, Hemoglobin, Hypertension, COPD, CKD, IHD, Beta Blocker, ACE-I, ARB, Diuretics, Ejection Fraction Category.

The SOFA score was significantly associated with in-hospital mortality. The odds ratios for 1-point increase in the SOFA score were 1.86 (95% CI 1.68–1.96) and 1.627 (95% CI 1.523–1.737) for in-hospital and 30-day mortality respectively (Table 2). The odds ratios for in-hospital mortality and 30-day mortality remained statistically significant after adjustment for demographic, laboratory and echocardiographic data with odds ratio of 1.874 (95% CI 1.719–2.04) and 1.657 (95% CI 1.541–1.781) respectively (Table 2).

The Kaplan–Meier curves for the distribution of time to death according to SOFA score categories are shown in Fig. 4, higher SOFA score is associated with increased risk of 30-day mortality log-rank < 0.0001.

Except for the respiratory component, all SOFA score components, tested as ordinal variables, had a statistically significant direct dose–response relationship with in-hospital and 30-day mortality (Table 3).

**Table 3:** The association between each SOFA individual component with in-hospital and 30 day mortality.

System	In hospital mortality odds ratio (95% CI)	P value	30 Day mortality odds ratio (95% CI)	P value
Neurologic	2.024 (1.777–2.305)	< 0.001	1.786 (1.57–2.02)	< 0.001
Hemodynamic	6.213 (4.852–7.957)	< 0.001	4.06 (3.325–4.95)	< 0.001
Respiratory	1.140 (0.993–1.308)	0.063	0.943 (0.841–1.057)	0.31
Liver	1.501 (1.194–1.886)	< 0.001	1.458 (1.197–1.776)	< 0.001
Renal	1.512 (1.336–1.711)	< 0.001	1.42 (1.278–1.579)	< 0.001
Platelets	1.513 (1.210–1.892)	< 0.001	1.4 (1.151–1.706)	< 0.001

**Figure 5:**
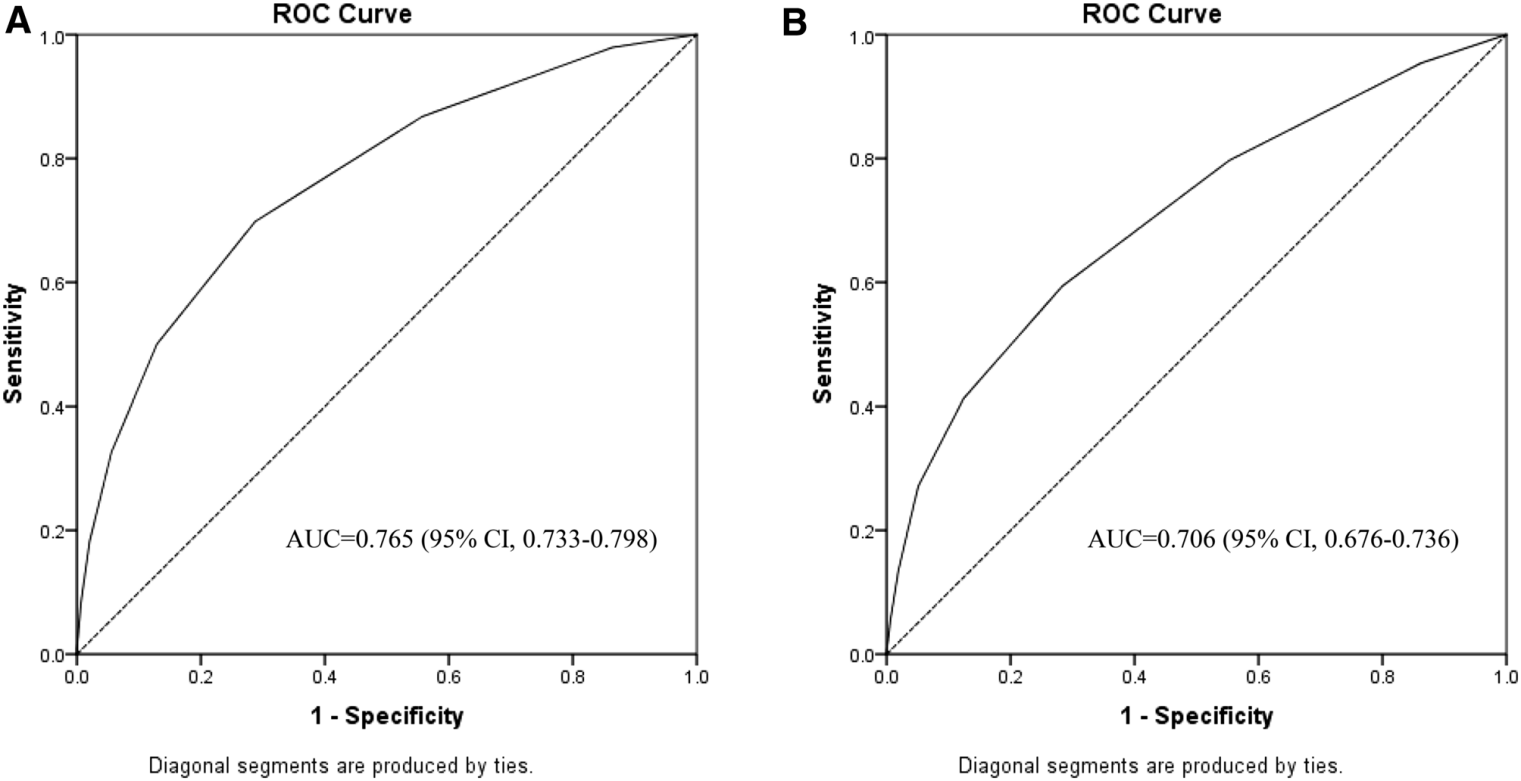
Area under the receiver operating characteristic curve (AUC) for predicting in-hospital mortality (**A**) and 30-day mortality (**B**) based on SOFA score.

The area under the ROC curves for the SOFA score were 0.765 (95% CI 0.733–0.798) for in-hospital mortality and 0.706 (95% CI 0.676–0.736) for 30-day mortality (Fig. 5).

Moreover, the SOFA score maintained its predictive accuracy when assessed as an ordinal variable of 4 risk categories, the AUC for were 0.752 (95% CI 0.719–0.785) and 0.695 (95% CI 0.665–0.725) for in-hospital mortality and 30-day mortality respectively.

**Figure 6:**
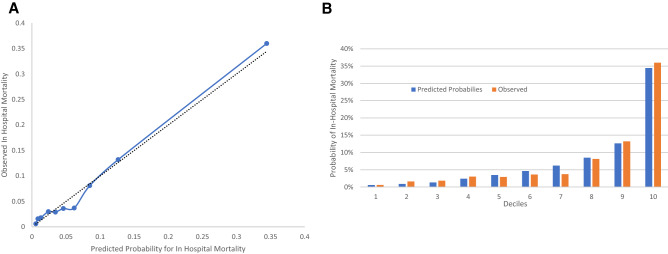
A. Observed vs predicted probability of in hospital mortality among predicted risk deciles. In (**A**) the dashed line is the identity line. The solid line represents the regression line. (**B**) Predicted probability vs observed probability of in hospital mortality.

Inspection of the predicted probability of in hospital mortality to the observed probability of in hospital mortality within the 10 deciles of predicted risk shows that overall, calibration appears reasonable in our population (Fig. 6).

Table 4 demonstrate the sensitivity, specificity, positive predictive value (PPV) and negative predictive value (NPV) of each SOFA score cutoffs. The optimal cutoff was SOFA score > 2 with sensitivity 69.83% and specificity 71%.

**Table 4:** Sensitivity, specificity, PPV, NPV at various threshold of the SOFA Score.

Cutoff	Sensitivity	Specificity	PPV (%)	NPV (%)
**Values for each SOFA score cutoff categories**				
> 0	97.93	13.61	8.4	98.8
> 1	86.78	44.27	11.2	97.6
> 2	69.83	71.21	16.4	96.7
> 3	50.00	87.09	23.9	95.6
> 4	32.64	94.42	32.1	94.5
> 5	18.18	97.96	41.9	93.7
> 6	7.85	99.40	51.4	93.0
> 7	3.31	99.77	53.3	92.7
> 8	0.83	100.00	100.0	92.6

*PPV* positive predictive value; *NPV* negative predictive value.

The SOFA score was compared with other validated risk scores; GWTG-HF, ADHERE and the Framingham risk score. Complete data were available for 2725 (84%) of the study cohort. The SOFA score demonstrated similar predictive accuracy compared to the GWTG-HF score in predicting in hospital and 30 day mortality, the AUC for predicting in hospital mortality were 0.733 (95% CI 0.757–0.789) and 0.752 (95% CI 0.735–0.768) respectively (P > 0.05). The SOFA score demonstrated superior predictive accuracy compared to the ADHERE and Framingham risk scores, the AUC for in hospital mortality were 0.591 (95% CI 0.572–0.609) and 0.501 (95% CI 0.482–0.519) respectively (P < 0.05) (Table 5).

**Table 5:** Comparison of the SOFA score to other risk scores (n = 2725).

Risk scores	In Hospital Mortality	P*	30 Day mortality	P*
	AUC (95% CI)		AUC (95% CI)	
SOFA	0.773 (0.757–0.789)	–	0.703 (0.686–0.720)	–
GWTG-HF	0.752 (0.735–0.768)	0.382	0.735 (0.718–0.751)	0.126
ADHERE	0.591 (0.572–0.609)	< 0.001	0.584 (0.565–0.602)	< 0.001
Framingham	0.501 (0.482–0.519)	< 0.001	0.526 (0.507–0.545)	< 0.001

*P value for the difference in AUC of the SOFA score and the other scores (DeLong test).

Furthermore, the predictive accuracy of the SOFA score was assessed according to heart failure type; Heart Failure with preserved ejection fraction (HFpEF), Heart Failure with reduced ejection (HFrEF) fraction and for patients with missing data of ejection fraction. The AUC was 0.791 (95% CI 0.743–0.839), 0.765 (95% CI 0.638–0.891) and 0.743 (95% CI 0.697–0.79) respectively.

The predictive accuracy of the SOFA score was assessed after splitting the cohort into early 5 (2008–2013) years and late 5 years (2014–2018). The AUC for in hospital mortality was 0.778 (95% CI 0.737–0.819) and 0.743 (95% CI 0.69–0.796) respectively. The AUC for 30 day mortality was 0.725 (95% CI 0.687–0.763) and 0.676 (95% CI 0.629–0.724) respectively.

**Figure 7:**
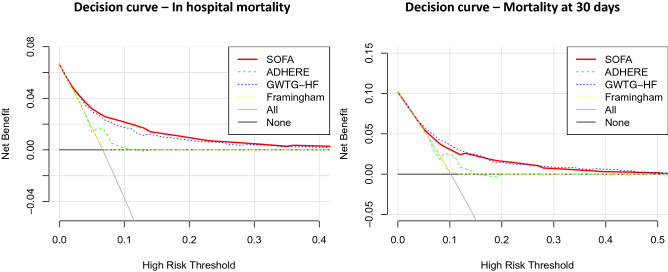
Decision curves for the SOFA score prediction of mortality . The net benefit (y-axis) of using the prediction model to guide clinical decision is plotted in relation to assuming that no one is at risk (all negative), that all are at risk (all positive). In-hospital mortaltiy and 30 day mortality are calculated based on the SOFA, GWTG-HF, Framingham and ADHERE score.

The decision curve analysis demonstrated positive net benefit of using the SOFA for decision thresholds between 5 and 45% in short term mortality (Fig. 7). The SOFA score had similar net benefit to the GWTG-HF score and higher net benefit than the ADHERE and the Framingham score across the range of risk thresholds.

## Discussion

The current analysis assessed the prognostic value of SOFA score in acute decompensated heart failure and found a direct correlation between short term mortality and the severity of SOFA score. The higher the SOFA score, the more increased mortality.

Despite age, demographic and comorbidities differences among the different SOFA categories, the SOFA score remained an independent predictor of in-hospital and 30-day mortality.

The predictive performance for in-hospital mortality of SOFA score was of a good predictive value (AUC, 0.765), similar to the predictive performance the studies in sepsis (AUC, 0.753)^11^.

Jentzer et al. demonstrated higher performance of the SOFA score in cardiac intensive care units (AUC, 0.83)^14^, however the study included patients admitted with a variety of critical care settings (myocardial infarction, arrhythmia, respiratory failure and sepsis) and patients with higher SOFA score severity regardless of the presence of cardiac disease.

A recent study by Aoyama et al. demonstrated good discriminative performance of the SOFA score in predicting long term mortality in ADHF (AUC, 0.689)^21^. However, the aforementioned study had small number of participants and included 15% patients with suspected sepsis that might lead to overestimation of SOFA predictive performance.

As expected, ADHF patients with high SOFA score have increased risk of mortality; as they have already consumed the hemodynamic and neurohormonal compensatory mechanisms, with subsequent severe systemic hypoperfusion, that impairs the function of the major components of the SOFA score; such as brain, kidney, liver and hematopoietic systems.

One of the interesting findings is that the respiratory component has low predictive capability for short-term mortality. This might be explained by the fact that ADHF presentation is dominated by volume overload and pulmonary congestion which reflects “wet and warm” patients, this presentation usually is relieved by intensified diuretic therapy^22^. On the other side of the spectrum are the “wet and cold” patients with organ dysfunction that prognosticate poorer outcome^16^.

As shown in previous studies, the hemodynamic component of the SOFA score was the strongest predictor of short-term mortality, those patients suffer from cardiogenic shock that is associated with poor prognosis. The rest of the SOFA score components (hemodynamic, renal, central nervous system, coagulation and liver) present a more severe decompensation with systemic hypoperfusion that is difficult to control.

Our study results suggest that the SOFA score has good discriminative performance for predicting short term mortality. The optimal cutoff according to Youden index is SOFA score above 2 points, yet this cutoff is far from optimal with sensitivity and specificity around 70%, and misclassified 2.2% as low risk patients who will eventually die, and misclassified 26% of patients as high risk without eventually dying. Our results regarding SOFA score are similar to various risk scores in ADHF setting, where the usefulness for the individual patient is far from optimal and should be implemented as a complementary tool for clinical expertise and judgement^23^.

The SOFA score might be integrated into the hospital computerized system, the computer will automatically calculate the score for patients admitted with ADHF, and can alert physicians to patients with SOFA score above 2 for the possibility of impending organ failure, and the need for intensive care unit and preparation for advanced heart failure team and utilities.

The SOFA score was compared to other validated scoring systems in heart failure, the SOFA demonstrated similar predictive accuracy compared to the GWTG-HF. One advantage of the SOFA score over the GWTG-HF is the dynamic nature of the SOFA components, rather than fixed variables of the GWTG-HF; age, black race and comorbid COPD.

The SOFA score demonstrated superior predictive accuracy compared to the ADHERE and the Framingham scores.

The SOFA score has a number of advantages compared to other scores that have also predictive capabilities. First of all, the score components are simple to calculate, available, affordable and known to critical care physicians. Secondly the score is dynamic and can be used daily to assess prognosis and response to treatment in critical settings. Thirdly the SOFA score is validated for various critical care illnesses and can be universally applied in the intensive care units.

The major strength of this study is the large number of participants admitted to various department settings (Internal medicine, cardiology, general and intensive care units) with real life ADHF patients. Furthermore, our study excluded patients with sepsis or infection during hospitalization to enhance the study precision, compared to a previous study that included considerable proportion of patients with infection^14,21^ .

### Study limitations

First, this is a retrospective single center study, the demographic data and SOFA score components were collected and calculated retrospectively. All the SOFA score variables expect the GCS were collected with high precision, the GCS was estimated according to the protocol explained in the method section. Our method in assessing the GCS might have introduced some misclassification, however we presume that this non-differential misclassification would bias our results to the null. Furthermore, the study assessed the SOFA at single time point at admission, therefore we cannot conclude from this study about the prognostic value of daily calculation. Furthermore, a large proportion of the study cohort lacked brain natriuretic peptides (BNP) levels because they were admitted before the standard clinical utilization of BNP.

## Conclusion

Patients with ADHF that have a high SOFA score are at increased risk of short-term mortality. SOFA score can be easily applied as complementary tool to clinical expertise to identify high risk patients. Future prospective studies are needed to confirm our findings.

## Supplementary information

Supplementary Information.

## Data Availability

The data underlying this article will be shared on reasonable request to the corresponding author.
