# Supplementary material for: SOFA score and short-term mortality in acute decompensated heart failure
Source: Sci Rep. 2020 Nov 30;10:20802. doi: 10.1038/s41598-020-77967-2 (PMC7705654; doi:10.1038/s41598-020-77967-2)
Supplement: Supplementary file 1 — Supplementary Information. [file 41598_2020_77967_MOESM1_ESM.docx]

**SOFA Score and short-term mortality in acute decompensated heart failure**

Adi Elias MD^1*^, Reham Agbarieh BSc^2^, Walid Saliba MD MPH^2,3^, Johad Khoury MD^4^, Fadel Bahouth MD^5,6^, Jeries Nashashibi MD^7^, Zaher S. Azzam MD^1,2^

1. Internal Medicine Department B, Rambam Health Care Campus, PO Box 9602, Haifa 31096, Israel.
2. Bruce and Ruth Rappaport Faculty of Medicine**, Technion – Israel Institute of Technology,** Haifa, Israel
3. Department of Community Medicine and Epidemiology, Lady Davis Carmel Medical Center, Haifa, Israel.
4. Pulmonology Division, Lady Davis Carmel Medical Center, Haifa, Israel
5. Cardiology Department, Rambam Health Care Campus, Haifa, Israel;
6. Internal Medicine Department H, Rambam Health Care Campus, Haifa, Israel;
7. Internal Medicine Department D, Rambam Health Care Campus, Haifa, Israel;

Correspondence: Dr. Adi Elias, department of Internal Medicine B, Rambam Health Care Campus, Haifa, Israel , email: adi.elias@gmail.com , postal address: PO Box 9602, Haifa 31096, Israel. Tel: 97247771976; Fax: 97247773252.

Sources of support: none.

Disclosure of funding: none.

Potential conflicts of interest: none.

Number of tables 5, Number of figures 7.

Keywords: Heart Failure, SOFA, Prognosis, Mortality.

**Supplementary Material:**

Table 1. Calculating the SOFA score and individual organ sub-scores.

| Organ system | Score 0 | Score 1 | Score 2 | Score 3 | Score 4 |
| --- | --- | --- | --- | --- | --- |
| **Cardiovascular**: Mean arterial pressure (MAP, mmHg) and vasopressors | MAP≥70 | MAP<70 without vasopressors | Dopamine≤5 or any dobutamine | Dopamine >5, Epineprhine≤ 0.1 or Norepinephrine ≤0.1 | Dopamine >15, Epineprhine> 0.1 or Norepinephrine >0.1 |
| **Central nervous system:**  Glasgow Coma Scale (GCS) | 15 | 13-15 | 10-12 | 6-9 | ≤6 |
| **Coagulation**:  Plaetelet count x 10^3^ /mm^3^ | >150 | ≤150 | ≤100 | ≤50 | ≤20 |
| **Liver**: serum bilirubin (mg/dl) | <1.2 | 1.2-1.9 | 2-5.9 | 6-11.9 | ≥12 |
| **Renal**:  Serum creatinine (mg/dl) and urine output ml/day | <1.2 | 1.2-1.9 | 2-3.4 | 3.5-4.9 or Urine output<500 | ≥5 or urine output < 200 |
| **Respiratory**:  Arterial PO2/FIO2 | >400 | ≤400 | ≤300 | ≤200 | ≤100 |
